# Hospital Reports

**Published:** 1897-06-26

**Authors:** 


### HOSPITAL REPORTS.

### THE OXYGEN HOME.

We have received the first annual report of the Oxygen Home for the Treatment of Ulcers and Wounds by Oxygen Gas. We wish the name had been differently chosen and the object of the institution differently laid down. If there is any difficulty in obtaining admission into general hospitals for patients suffering from ulcers, a class of persons deserving of every commiseration, people are quite within their rights in founding an institution where they may receive such treatment as may be necessary for the cure of these unfortunates. But to found an institution for the carrying out of one form of treatment only is practically equivalent to condemning a considerable proportion of the patients to a treatment which may not be suited to their cases—unless indeed Dr. Stoker is prepared to maintain that all ulcers are best treated by the so-called oxygen method, a thesis which we think he will find it very difficult to support. In any case the title adopted by this institution is a misnomer, for according to the published reports other things besides oxygen are used in the treatment, and the cases in which oxygen alone was used did not do well. Till it is decided what is the active factor in producing the results claimed it seems a pity that the medical officer should be tied down to one sort of treatment. Nitrogen perchance might be better still!

